# Application of Multilevel Models in Dentistry

**Published:** 2017-11

**Authors:** Mohammad Javad Kharazifard, Kurosh Holakouie-Naieni, Mohammad Ali Mansournia

**Affiliations:** 1 PhD Candidate of Epidemiology, Department of Epidemiology and Biostatistics, School of Public Health, Tehran University of Medical Sciences, Tehran, Iran; 2 Professor, Department of Epidemiology and Biostatistics, School of Public Health, Tehran University of Medical Sciences, Tehran, Iran; 3 Assistant Professor, Department of Epidemiology and Biostatistics, School of Public Health, Tehran University of Medical Sciences, Tehran, Iran

**Keywords:** Multilevel Analysis, Multicenter Study, Clinical Trials, Dentistry

## Abstract

Multilevel analysis which was primarily introduced to deal with hierarchical data was later applied extensively for research in other fields of science and not only for nested data, but also for repeated measurements or clustered trials. This method of statistical analysis was applied in dental studies in the 1991 for the first time but despite its value for data analysis in dental studies, its application for dental studies remains limited until now. This manuscript reviews the applications of this method in dental studies.

## INTRODUCTION

Multilevel analyses are attempted to find correlations among variables that have a hierarchical or nested nature. They are aimed at eliminating the problems due to the shortcomings of traditional analyses in perceiving the correlations among variables at different levels [1]. This type of analysis was first introduced by Aitkin et al, [2] in the early 1980 for the purpose of educational assessment for nested data. It was later applied extensively for research in other fields of science and not only for nested data, but also for repeated measurements or clustered trials [3].

**Fig. 1:**
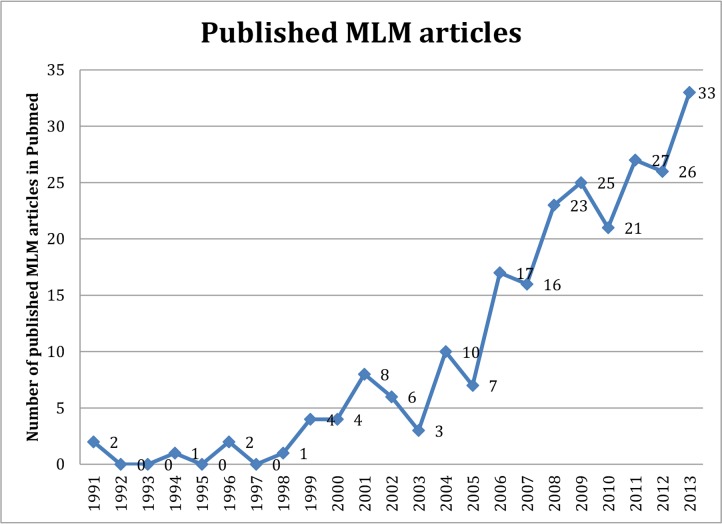
Number of dental articles with multilevel analysis (MLM articles) published in PubMed from 1991 to the end of 2013

**Fig. 2:**
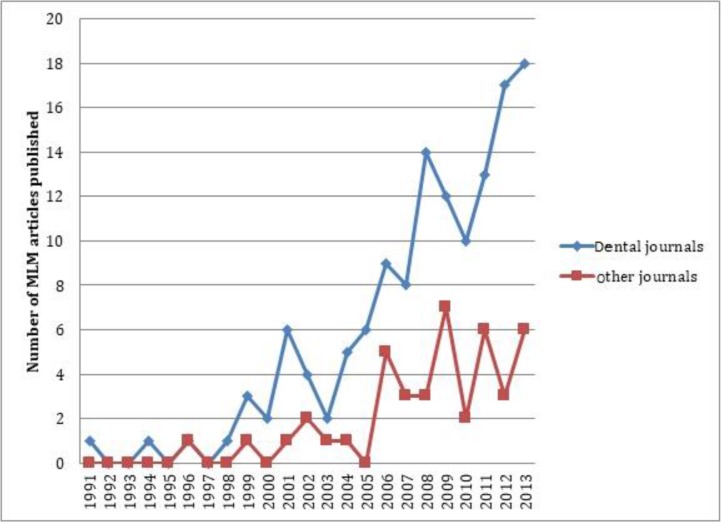
Number of dental articles published in dental and non-dental journals in PubMed from 1991 to the end of 2013 using multilevel analysis (MLM articles)

In dentistry, multilevel models were first applied in 1991 for the assessment of craniofacial growth curves for orthodontic purposes [4]. Although the nature of most data in dental research is compatible with such analyses, they have been rarely used in studies. Figure 1 shows the number of dental articles published in PubMed during 1991–2013 that used multilevel analysis. In the mentioned time period, multilevel analysis was used in only 233 articles; out of which, 42 had been published in non-dental and 191 in 45 dental journals. Number of dental and non-dental journals that published multilevel model dental papers in this time period is shown in Figure 2. A noteworthy issue is that of 191 papers published in dental journals, 155 had been published in specialty journals in the fields of periodontics, orthodontics and oral health and community dentistry and a total of 184 articles were in the mentioned three fields. Also, 91 articles had been published in six and 123 papers in 10 specific journals. Therefore, it seems that using multilevel analysis is by invitation and demand of reviewers and editors of specific journals familiar with this method rather than being the choice of researchers.

All that said, with the presumption of lack of familiarity of dental researchers with multilevel analysis and its applications despite the passage of many years since its introduction, this study sought to reintroduce this model by setting examples of its different applications in dental research.

Although the aim of this study is not providing the mathematical model of multilevel analysis or discussing its different types and characteristics, for the purpose of primary acquaintance, we offer a simplified definition of this model.

In unilevel (ordinary) regression model, the following formula is used to assess the effect of variable X on factor Y.

$${\text{Y}}_{\text{i}}=\beta 0+\beta 1{\text{X}}_{\text{i}}+{\text{e}}_{\text{i}}$$

Where β0 is a fixed amount, β_1_ is the slope in the conditional regression model for Y|X and e is the estimated error rate. If in this model, several X_j_ (from X_1_ to X_n_) are used instead of one independent variable, the model will be in the following form:

$${\text{Y}}_{\text{i}}=\beta_{0}+\Sigma \beta_{\text{j}}{\text{X}}_{\text{ij}}+{\text{e}}_{\text{i}}$$

Assuming that in studies with hierarchical data, a higher-level variable affects the lower-level ones, the equation can be written in such way that all elements in the equation are influenced by the upper level variable. It means that, for each amount of the higher level variable, the coefficients of the equation will change. In other words, instead of one equation, we will have a separate equation for each amount of the higher-level variable.

Assume that in your study, you want to assess the effect of oral hygiene instruction (ME) on plaque index (PI) of students. To simplify the model in this study, we only consider one covariate i.e. age. In this case, the suggested model for unilevel regression analysis would be as follows:

$${\text{Y}}_{\text{i}}=\beta_{0}+\beta_{1}{\text{age}+\beta }_{2}\text{ME}+{\text{e}}_{\text{i}}$$

If this study was carried out as clustered in different schools, in order to assess the effect of type of school in the model, for each of the β_0_, β_1_ and β_2_ coefficients in the multilevel equation, the effect of type of school variable (S) would also be taken into account as follows:

$$\begin{array}{l} \beta_{0}=\delta_{00}+\delta_{01}{\text{S}}_{\text{j}}+{\text{U}}_{0\text{j}}\\ \beta_{1}=\delta_{10}+\delta_{11}{\text{S}}_{\text{j}}+{\text{U}}_{1\text{j}}\\ \beta_{2}=\delta_{20}+\delta_{21}{\text{S}}_{\text{j}}+{\text{U}}_{2\text{j}} \end{array}$$

Thus, for each type of school, a different equation is created to assess the effect of type of intervention on PI of students at different ages. Such change in coefficients of the equation can well justify the difference in the efficacy of education in different schools. One advantage of this model is that by using a specific type of this model known as variance component model, we can find out the percentage of the changes of a dependent variable that occur at each level. In the aforementioned example, it can be found that how much of the change in PI is due to the individual changes and how much is due to differences between students of different schools [3].

### Hierarchical data:

In dentistry, similar to other fields of science, health-related outcomes and health or disease status occur as the result of the interaction effect between the individuals and their surrounding environment. These factors are known as the social determinants of health and in order to recognize them and evaluate their effect on oral health, multilevel models must be applied.

In the domains of oral health and community dentistry, social factors are important determinants of the risk of tooth decay. “There are several reasons why neighborhood environment may affect dental caries in negative and positive ways. Availability of, and access to, healthy foods may differ across areas, as may availability and access to dental care” [5]. Detecting the effect of these factors and their interaction with individual risk factors requires multilevel analysis.

Tellez et al, [5] in their study found that the likelihood of caries development was lower in areas with higher number of churches; whereas, risk of caries development increased in areas with higher number of grocery stores. However, in some investigations, despite the conduction of multilevel analysis, the effect of factors in higher than individual levels is found to be insignificant and the analysis eventually turns into a unilevel regression analysis [6]. Nonetheless, first the effect of social factors must be assessed in multilevel models because the magnitude of the effects of factors at the social level is variable in different communities and must be measured. Moreover, the effect of variables at both individual and social levels on developing hygiene habits like tooth brushing can be evaluated in such studies. For instance, a study conducted in Pretoria in 2009 demonstrated that the effect of school grade variable on frequency of tooth brushing decreased while the age increased from 11 to 15 [7].

Multilevel modeling has also been applied to estimate the effect of individual and social parameters on the likelihood of developing periodontal disease [8] or the prevalence of pain [9, 10]. These models are especially important when assessing the effect of social factors on diseases, in which environmental factors play a more serious role like fluorosis [11].

Both individual and social factors play a role in development of caries but despite the fact that all teeth are located in the same environment, the oral cavity, carious lesions occur in some of them and other teeth remain intact, at least by the time of examination. Such differences confirm the theory of the effect of some factors at a lower than individual level on development of caries. These variables can be related to the anatomical form of the teeth, their location in the jaw and dental arch, class of malocclusion and even presence of crowding; because the location of accumulated microbial plaque also affects the efficacy of plaque removal. Presence of such variables necessitates the use of multilevel models to study the behavior of tooth decay and investigate the effect of variables at lower than individual levels namely at the level of tooth or even tooth surface [12]. Barga et al. [13] used this statistical approach and evaluated the effect of different factors at the individual and tooth levels on presence of active caries in the occlusal surface of the primary teeth.

In studies related to periodontal disease, variables must be evaluated at a lower than individual level. Muller [14] in 2009 compared unilevel and multilevel regression models to assess the correlation of gingival thickness and width and demonstrated that although this correlation was not significant in unilevel model, multilevel model was capable of detecting it considering covariates at the individual and tooth levels. Moreover, the likelihood ratio of the model significantly increased; in other words, multilevel model had a higher model fitness. For the same reason, multilevel analysis is also recommended to assess implant treatment success rate [15].

For assessment of periodontal parameters such as pocket depth, data are evaluated at a level smaller than the tooth. For instance, periodontal pocket depth or clinical attachment level are usually measured and reported in six points around the teeth. Thus, for the assessment of periodontal status, we need to define a level lower than the tooth level, known as the site level. Axtellius et al, [16] in 1999 set a practical example and showed that taking into account the three levels of site, tooth and individual, using multilevel model is necessary for the assessment of pathogenesis and progression of periodontal disease.

In randomized clinical trials where a therapeutic or preventive intervention for periodontal disease is performed at the individual or tooth level, using multilevel models can be a suitable solution in order to indicate the effect of all variables at all levels taking into account the site specific nature of outcome variables [17–19].

In clinical trials where the operator is more than one person, another level can be defined and added to the model as the operator level. By doing so, the effect of operator’s skills and expertise can be assessed as well [20].

### Multistage sampling and clustered trials:

In descriptive studies with multistage sampling, subjects within clusters show a more similar response than those outside the cluster due to the similarities in demographic and environmental variables. As the result, in spite of equal sample size, variance in this method of sampling is different from that of single-stage sampling. In order to solve this problem, it should be determined that what percentage of the changes is related to the cluster effect. For this purpose, variance inflation factor is calculated taking into account the intraclass correlation coefficient of specimens inside a cluster. To put it simply, the greater the degree of similarity of samples inside a cluster compared to those out of the cluster, the higher the variance inflation factor. The effect of each level on the rate of variance can be detected using a basic multilevel model called the variance component model. Moreover, using multilevel models, the effect of each variable at the individual (i.e. demographic variables) and cluster (i.e. place of residence or neighborhood) levels on the response variable can be assessed [21].

In randomized clustered trials where the intervention is performed at a level other than the individual level, samples inside a cluster are expected to show a more similar response than samples in a different cluster. In such studies, the effect of intervention is overestimated compared to individual-based trials due to the aforementioned intragroup similarity. To avoid such bias, multilevel analysis should be applied and two levels namely individual and cluster levels must be defined. Yekaninejad et al. [22] evaluated the effect of oral hygiene instruction at school on the frequency of tooth brushing using multilevel analysis.

In addition to qualitative and quantitative data analysis, multilevel models can also be used for survival data analysis. Multilevel survival data models can also be used for the assessment and comparison of the longevity of dental restorations; for example, assessment of the durability of amalgam restorations of different teeth in the same patient by considering the tooth and dependent factors as level 1 and the individual as level 2 [23]. Furthermore, Stephenson et al. [24] applied this model to compare two competing risks of primary teeth extraction due to caries and exfoliation at two levels of tooth and individual and to detect the factors affecting severe caries of primary teeth. Gilthorpe et al. [25] assessed the survival of amalgam restorations using a three-level model. In their study, similar to the one by Kopperud et al, [23] two levels of individual and tooth were considered in the model. However, as the lowest level, the effect of restoration replacement variable was also assessed. It means that the model offered separate equations for the primary and secondary restorations.

### Repeated measures:

One application of multilevel models is for the analysis of repeated measures in longitudinal studies. In such studies, a measurement or index is assessed in several visits or follow up sessions during a specific time period. One response is obtained at each time point. Assessment of these changes in a specific time period can be done using repeated measures analysis. For instance, repeated measures ANOVA can be applied for analyzing continuous, quantitative variables. Multilevel models have been introduced as a tool for such variables. In these types of models, measurements at each time point are considered as level 1 and the individual or specimen is considered as the level 2 of the analysis.

In dentistry, like in other fields of biomedical sciences, longitudinal studies are carried out with frequent assessment time points and follow ups. These studies have a wide spectrum in different types of methodologies ranging from pure descriptive to multiple follow up clinical trials or even multi-stage in vitro studies.

Longitudinal assessment of growth during a specific time period is an example of descriptive studies. In these studies, growth curve can be estimated based on anthropometric measures. For almost 10 years in the late 20^th^ century, the only application of multilevel analysis in dental research was limited to the estimation of craniofacial growth equations [26]. These equations were fitted based on age variable and in the form of curves with fifth and fourth order equations of age variable rather than a linear equation [26,27]. Although at first studies merely estimated the growth curves, later studies investigated the effect of demographic variables as well [28]. Also, Barrera et al. [29] used this model to assess changes in the masticatory function during the period of growth and development.

These models can also be used in clinical trials with multiple follow up sessions. For instance, this model was applied to compare root coverage in two methods of coronally advanced flap in a split-mouth, randomized controlled trial during 14 years [30].

In some longitudinal studies, data at each time point have a hierarchical nature. In such cases, time, as the lowest level, can be entered into the multilevel regression model [31]. Gilthorpe et al. [32] conducted a longitudinal study on periodontal patients to test the accuracy of two theories regarding the mechanism of periodontal disease progression. They applied a 4-level (repeated measurement, site, tooth, subject) multilevel model to assess the trend of changes in pocket depth.

The understudy sample can be human, animal or even laboratory experiments, and multilevel models can be used as long as several follow ups are made. A few recent studies have used multilevel models for the analysis of data with several follow up sessions in animal models. For instance, Liu et al, [33] in 2010 used this method of analysis to compare the effect of continuous and intermittent load on suture expansion in New Zealand white juvenile male rabbits at different time points. This method has also been used in in vitro studies to compare the microbial colony count in the water flow of dental units at different time points [34].

In laboratory study designs or repeated measures, crossover or paired-data trials, multilevel models can be applied based on the assumption of repeated data of each specimen at two time points or two different areas of a specimen. Tang et al. [35] used this method of analysis to compare the masticatory function in two- and four-implant supported overdentures. In a crossover, randomized trial, they fabricated two types of overdentures for patients and compared the masticatory function of patients in the two groups at three different time points with five foods. In this three-level model, the tested food products comprised level 1, time of assessment comprised the 2^nd^ level and the overdenture design was the 3^rd^ level. A similar approach was used in a split-mouth animal study [36]. In many cases, both multilevel and unilevel repeated measure models can be used for the analysis of repeated data. Even in many cases, unilevel repeated measure analyses are preferred to multilevel models due to their easy application and interpretation. However, the weakness of unilevel model is the necessity of presence of data at all follow ups. If a sample does not show up in one follow up, that specimen must be totally excluded from the analysis or that specific data must be imputed with specific methods. Applicability of multilevel model in such cases is a strength point of this model for the analysis of repeated data. Furthermore, multilevel models can be easily applied to different types of variables; while unilevel models have some limitations for ordinal or count data variables. Multilevel models are more suitable for the assessment of the effect of explanatory variables and the interaction of outcomes compared to single level analysis [3, 37].

In cases where several response variables are derived from a series of specimens, considering the fact that all responses are derived from a single series of specimens, the hypothesis of independence of data is rejected and separate analysis of each variable increases α error. Thus, multiple response studies can be considered as a variant of repeated measures and multilevel models can be applied for data analysis in these cases. In such studies, the response variables comprise the level 1 and the study samples comprise the level 2. For instance, Pereira et al. [38] used this model to assess the correlation of detectable plasmatic HIV viral load with the accumulation of 35 microorganisms at the subgingival area in AIDS patients.

### Diagnostic studies and validity measurements:

In studies assessing clinical diagnostic techniques, particularly for caries detection, data have a hierarchical nature; because when comparing diagnostic methods, whether the aim is to assess validity or reliability, assessments are done on single series of samples; which makes the comparison of different techniques difficult with the conventional analytical methods. In caries detection clinical studies, the reproducibility of caries detection can be evaluated at the three levels of individual, tooth and surface [39].

### Systematic reviews:

In systematic reviews, researchers try to combine the results of different studies in order to draw a general conclusion. Thus, considering the two levels of sample and study, multilevel models can be applied for data analysis. This method has one main advantage over the conventional meta-analysis methods; that is, studies reporting different results do not have to be necessarily excluded in case of heterogeneity of results; because all studies can be presented in a bi-level model [40].

In a systematic review aiming to assess orthodontic bond strength in-vitro, since the primary studies had been conducted by a few specific authors, the three-level model was used for data analysis [41].

## CONCLUSION

Introduction of multilevel models, due to their extensive applications, has revolutionized the analysis of study results in the fields of social sciences as well as medical sciences. In medicine, these models can be applied to all types of data retrieved from a wide range of studies from epidemiologic and descriptive to clinical trails and even laboratory experimental studies. By the advances in different multilevel models, they can now be applied to different outcome variables with linear regression, logistic regression [31], Poisson [42], negative binomial [43] and even survival data [23] models.

At first, limited software programs were introduced for these analyses; but, at present, the majority of multilevel analyses can be performed by some software programs like SAS and R. Simpler multilevel models can even be accessed in conventional statistical software programs like SPSS and STATA. The noteworthy issue here, is the introduction of these methods, their principles, applications and advantages for data analysis and particularly presenting models that better fit the reality of biomedical phenomena than single level models.
